# Metabolic remodeling induced by mitokines in heart failure

**DOI:** 10.18632/aging.102247

**Published:** 2019-09-09

**Authors:** Jiahao Duan, Zijun Chen, Yeshun Wu, Bin Zhu, Ling Yang, Chun Yang

**Affiliations:** 1 Department of Cardiology, The Third Affiliated Hospital of Soochow University, Changzhou 213003, China; 2 Department of Critical Care Medicine, The Third Affiliated Hospital of Soochow University, Changzhou 213003, China; 3 Department of Anesthesiology, Tongji Hospital, Tongji Medical College, Huazhong University of Science and Technology, Wuhan 430030, China

**Keywords:** heart, metabolism, mitohormesis, peptides, retrograde signaling

## Abstract

The prevalence rates of heart failure (HF) are greater than 10% in individuals aged >75 years, indicating an intrinsic link between aging and HF. It has been recognized that mitochondrial dysfunction contributes to the pathology of HF. Mitokines are a type of cytokines, peptides, or signaling pathways produced or activated by the nucleus or the mitochondria through cell non-autonomous responses during cellular stress. In addition to promoting the communication between the mitochondria and the nucleus, mitokines also exert a systemic regulatory effect by circulating to distant tissues. It is noteworthy that increasing evidence has demonstrated that mitokines are capable of reducing the metabolic-related HF risk factors and are associated with HF severity. Consequently, mitokines might represent a potential therapy target for HF.

## INTRODUCTION

Heart failure (HF) is an urgent global public health problem because of its high morbidity, high mortality, and high rehospitalization rate [1]. The proportion of HF may rise [2] because of prolonged life, increased prevalence of risk factors, and improved survival rates from other cardiovascular diseases (CVDs) [3, 4]. Currently, advancements in the treatment of ischemic and valvular heart disease have greatly improved the survival rates; however, residual cardiac dysfunction and postoperative complications lower the quality of life, ultimately leading to the development of HF [5]. In addition, therapeutic strategies for HF rehospitalization are mainly limited to symptom reduction, such as regulation of the neuroendocrine function and reduction in heart rates. These strategies aim to unburden the heart and reduce the myocardial oxygen demand in order to rebalance energy production and consumption at a low efficacy as well as prevent or slow ventricular remodeling [6]. Despite symptom reduction, the patients’ quality of life and long-term prognosis may be favorable [5]. Consequently, therapeutic strategies that improve myocardial contractility and pumping function without causing adverse effects similar to those caused by inotropic drugs are required in clinical practice [7]. Unfortunately, stem cell therapy for HF does not appear promising [8, 9], and novel therapies are under research.

**Figure 1:**
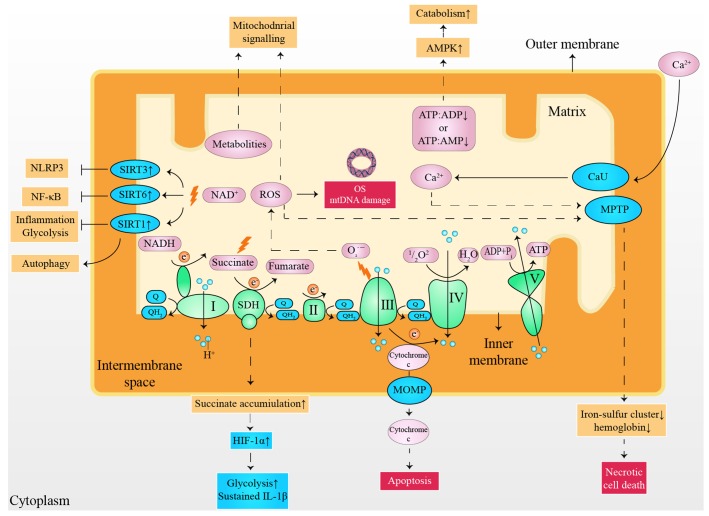
**Small molecules arising from the mitochondria served as cellular and systemic signals.** ATP: adenosine triphosphate; ADP: adenosine diphosphate; AMP: adenosine monophosphate; AMPK: 5' adenosine monophosphate-activated protein kinase; CaU: calcium uniporter; e^-^: electron; HIF-1α: hypoxia-inducible factor 1-alpha; IL-1β: interleukin 1 beta; mtDNA: mitochondrial deoxyribonucleic acid; MOMP: mitochondrial outer membrane permeabilization; MPTP: mitochondrial permeability transition pore; NLRP3: nucleotide-binding oligomerization domain-like receptors pyrin domain containing 3; NF-κB: nuclear factor kappa-light-chain-enhancer of activated B cells; NADH: nicotinamide adenine dinucleotide; OS: oxidative stress; Q: ubiquinone; QH_2_: ubiquinol; ROS: reactive oxygen species; SIRT1: sirtuin1; SIRT3: sirtuin3; SIRT6: sirtuin6; SDH: succinate dehydrogenase.

Although a normal heart accounts for only about 0.5% of the total mass of human body, the proportion of cardiac adenosine triphosphate (ATP) consumed each day reaches 8% [5]. Moreover, energy consumption is increased exponentially under cardiac stress [10]. Consequently, insufficient myocardial energy supply [11], substrate utilization disorder [12], and oxidative stress (OS) [13] are considered to be responsible for the progress of HF. However, it appears difficulty to treat HF from a metabolic perspective, considering the flexibility of cardiac substrate metabolism [14] and the complex metabolic network [15]. It has been validated that small molecules derived from mitochondria have capability to serve as cellular and systemic signals, such as adenosine monophosphate (AMP), adenosine diphosphate (ADP), reactive oxygen species (ROS), Ca^2+^, NAD^+^, cytochrome c, succinate and metabolites (Figure 1) [16–19]. It is noteworthy that moderate mitochondrial dysfunction or stress reduce risk factors of HF [20], partly owing to the metabolic regulation of mitokines produced via cell non-autonomous responses [21, 22]. In detail, fibroblast growth factor 21 (FGF21), growth differentiation factor 15 (GDF15), adropin, and irisin are encoded and released by the nucleus, regulating inter-tissues metabolism [22, 23]. In contrast, mitochondria-derived peptides (MDPs) and mitochondrial unfolded protein response (UPR^mt^) are encoded by mitochondrial genomes that upregulate the expression of chaperones, proteases, and mitochondrial biogenesis by acting as retrograde signaling [24, 25]. Furthermore, mitokines are also capable of improving cell metabolism via indirect methods [26–28], such as inhibition of inflammation, alleviation of OS damage, reduction of autophagy, and delay in cellular aging (Table 1). This review outlines the significance of the mitochondria for cardiac function maintenance, highlighting the metabolic characteristics in healthy and diseases heart with a summary of the possible roles and mechanisms of mitokines in CVDs. Finally, we discuss the possibilities and challenges of mitokines as a potential target for HF and indicate important research areas.

**Table 1:** The role of mitokines in heart failure.

**Mitokine**	**Encoded**	**Signaling pathway**	**Function**	**Application**
Nucleus-derived				
FGF21 [81–100]	FGF21 gene	Akt1-GSK-3β-caspase3; ERKs; Ucp3; ATF4	Anti-OS; Autophagy protection	HF prevention
GDF15 [120–129]	GDF15 gene	GFRAL	OXPHOS improvement	HF biomarker
Adropin [130–136]	ENHO	GPCR-MAPK-PDK4; VEGFR2-ERK1/2	Endothelial protection	HF biomarker
Irisin [137–142]	FNDC5 gene	AMPK-ULK1	Autophagy protection	HF biomarker
Mitochondria-derived				
Humanin [106–112]	Mt 16S rRNA	STAT3	Anti-OS; Anti-apoptosis OXPHOS improvement	HF prevention
SHLPs [104, 113]	Mt 16S rRNA	STAT3; ERKs	Similar to humanin	HF prevention
MOTS-c [114–119]	Mt 12S rRNA	folate-AICAR-AMPK; MAPKs; NF-κB	Anti-inflammatory; Endothelial protection	HF prevention
UPR^mt^ [143–159]	bZIP domain	ATFS-1; ATF5; SIRT3-AMPK	Protein regulation; Anti-OS; OXPHOS inhibition	HF biomarker; Therapeutic target

Abbreviations: AMPK: 5′-AMP-activated protein kinase; AICAR: minoimidazole-4-carboxamide ribonucleotide; ATFS-1: activating transcription factor associated with stress-1; ATF: activating transcription factor; bZIP domain: the basic leucine zipper domain; ERKs: extracellular signal-regulated kinases; FGF21: fibroblast growth factor 21; FNDC5: fibronectin type III domain-containing protein 5; GDF15: growth differentiation factor 15; GSK: glycogen synthase kinase; GFRAL: glial cell-derived neurotrophic factor (GDNF) family receptor α-like; GPCR: G protein-coupled receptor; HF: heart failure; MOTS-c: mitochondrial open reading frame of the 12S rRNA-c; mt: mitochondrial; MAPK: mitogen-activated protein kinase; NF-κB: nuclear factor kappa-light-chain-enhancer of activated B cells; OS: oxidative stress; OXPHOS: oxidative phosphorylation; PDK4: pyruvate dehydrogenase lipoamide kinase isozyme 4; rRNA: ribosomal ribonucleic Acid; SHLPs: small humanin-like peptides; STAT3: Signal transducers and activators of transcription 3; SIRT3:sirtuin3; UPR^mt^: mitochondrial unfolded protein response; Ucp3: Mitochondrial uncoupling protein 3; ULK1: Unc-51 like autophagy activating kinase1; VEGFR2: vascular endothelial growth factor receptor 2.

## Characteristics of myocardial metabolism

The mitochondria are critical for the maintenance of adult cardiac function, given its potent capacity to produce ATP, ability to regulate Ca^2+^, and the ability to induce myocardial pathological inflammatory responses and apoptosis [29, 30]. The mitochondria account for approximately 25%–30% of the volume of cardiomyocytes, widely distributed in the subsarcolemmal, perinuclear, and intramembranous regions [5]. Mitochondria support > 95% of the myocardial ATP demand through oxidative phosphorylation (OXPHOS) [14]. Mechanistically, glycolysis, fatty acid (FA) β-oxidation (FAO), and tricarboxylic acid cycle are the main sources of H^+^ and electrons. Nicotinamide adenine dinucleotide (NADH) and reduced flavin adenine dinucleotide (FADH_2_) transfer H^+^ and electrons to the electron transfer chain (ETC) composed of complex I to complex IV [31]. Correspondingly, the energy released by the protons is pumped from the mitochondrial matrix into the intermembrane space across the inner membrane [32]. Considering the high impermeability of the mitochondrial inner membrane, a chemical gradient (ΔpH) and an electrical gradient (ΔΨm) are built up across the inner membrane [33]. The proton motive force (PMF), the collective name of ΔpH and ΔΨm, drives the phosphorylation of ADP to form ATP at F_0_F_1_-ATP synthase, along with the generation of a small amount of ROS [32].

Myocardial metabolism has its own characteristics. First, the metabolic substrate of a healthy heart is flexible. FA (60%–90%) and ketone bodies (10%–40%) are the main substrates under physiological conditions [12]. Despite a higher utilization efficiency of glucose, glucose merely accounts for 5% of the cardiac oxidation [34]. In-vivo and in-vitro experiments have demonstrated that glucose metabolism is restrained by FAO and is related to dietary and physical activity [35]. However, glucose oxidation instead of FAO takes charge of myocardial metabolism during cardiac overload [12]. Furthermore, ketone bodies might become the main substrate for fasting or poorly controlled diabetes. Second, myocardial metabolism has a specific regulation mechanism. 5′-AMP-activated protein kinase (AMPK) activates along with the increased AMP content during ATP shortage [36]. ATP content is increased via AMPK-mediated inhibition of ATP consumption, promotion of FA, and oxidation of glucose [37]. In addition, peroxisome proliferator-activated receptors (PPARα) are validated to regulate the long-term cardiac metabolism [14]. PPARα is capable of upregulating the transcription of genes related to FA uptake and OXPHOS, enhancing the cardiac oxidation ability. Its activation depends on PPARγ co-activator 1α (PGC1α) or PGC1β [14, 38]. Third, the buffer between the mitochondria and the cytoplasm guarantees heart energy supply during cardiac stress. Excess ATP shifts the phosphate bond to creatine via the action of creatine kinase (CK) to form phosphocreatine (PCr) that is rapidly transferred to the cytoplasm [14]. PCr has the ability to transfer the phosphate bond to ADP, forming ATP again during the first 7 *s* of cardiac stress [39]. These mechanisms ensure ATP supply during a sudden cardiac attack [40].

**Figure 2:**
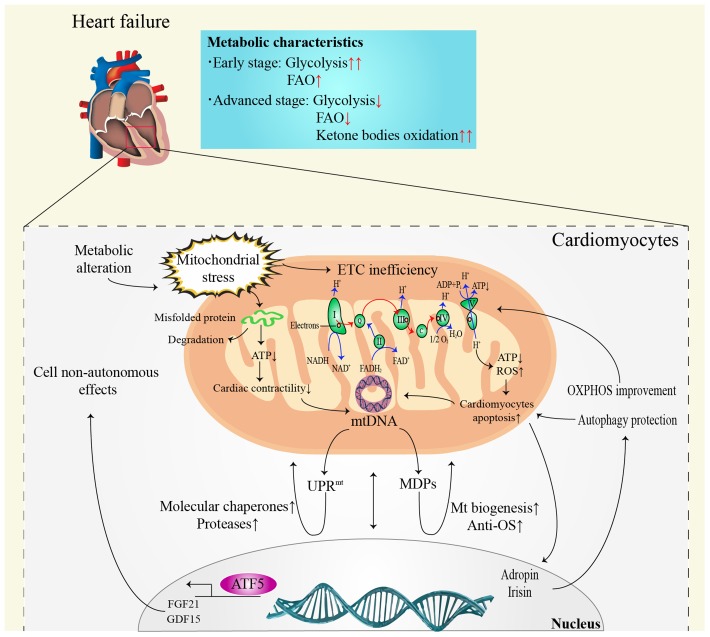
**Communication between mitochondria and nucleus in heart failure.** ATP: adenosine triphosphate; ADP: adenosine diphosphate; ATF5: activating transcription factor 5; DNA: deoxyribonucleic acid; ETC: electron transfer chain; FAO: fatty acid β-oxidation; FADH_2_: reduced flavin adenine dinucleotide; FGF21: fibroblast growth factor 21; GDF15: growth differentiation factor 15; Mt: mitochondrial; MDPs: mitochondria-derived peptides; NADH: nicotinamide adenine dinucleotide; OS: oxidative stress; OXPHOS: oxidative phosphorylation; ROS: reactive oxygen species; UPR^mt^: mitochondrial unfolded protein response.

The most prominent metabolic change in HF is the conversion of FAO to hypoxic carbohydrate metabolism, such as glycolysis [41]. It is noteworthy that the alterations in myocardial metabolism depend on the HF stage (Figure 2). In the early stage of HF, FAO remains unchanged or slightly elevated [42], while glucose uptake and glycolysis increase significantly [43]. However, both FAO and glucose metabolism efficiency decrease in advanced or end-stage HF [42]. It is noteworthy that ketone bodies seem to become the main metabolic substrate in advanced stage HF [41, 44]. The oxidation of ketone bodies has been validated to improve the efficiency of myocardial metabolism and cardiac function in HF [45]. Despite the positive function, the long-term impacts of ketone body metabolism on HF patients still need to be elucidated.

## Novel insights into the high-risk factors of HF

### Obesity and insulin resistance (IR)

It is clear that IR plays an essential role in atherosclerosis and hypertension [46]. Type 2 diabetes and obesity are independent risk factors that increase the morbidity of HF [47]. However, whether obesity and IR induce HF remains controversial. Moderate IR might be beneficial to the heart by exerting a protective effect against the damage caused by excessive accumulation of myocardial substrate [14]. In addition, it seems that the alteration of substrate rather than a high-fat diet (HFD) is more likely to increase metabolic stress and induce cardiac dysfunction [48]. These differences might be attributable to the dietary styles, duration of therapy, and species. Although obesity has been validated as an independent risk factor for HF [49], overweight is related to lower HF mortality [50]; this is called the obesity paradox. It is hypothesized that overweight and obesity have the potential to exert protective effects in the aged and those with chronic diseases [51, 52]. Obesity paradox was first observed in 1999 in patients undergoing hemodialysis who were overweight and obese [53]. More importantly, obesity paradox was also found in patients with HF [54, 55]. Hemodynamic changes [56] and cytokine responses [57] in HF are associated with impaired gastrointestinal function [58], anorexia [56], and hypermetabolism [59], contributing to the development of cardiac cachexia. Consequently, patients with HF commonly exhibit weight loss [60]. Furthermore, obesity is capable of increasing the muscle mass in patients who have HF with reduced ejection fraction (HFrEF) [61]. In contrast, 239 prospective studies have disproved the obesity paradox, suggesting that obesity and overweight are closely related to higher all-cause mortality [62]. However, there remains considerable debate whether obesity paradox contributes to cardiac remodeling and the risk of HF with preserved ejection fraction (HFpEF) [63]. The discrepancy might be related to age, sex and other comorbidities [64]. Objectively, obesity increases the morbidity and mortality of HF. However, it appears reasonable for patients with advanced HF to gain weight properly to offset the weight loss caused by HF. Further optimized studies that aim to assess this difference are urgently required.

### Oxidative stress (OS)

Conventionally, the abnormal release of ROS induced by OS is considered the main cause of cell senescence and apoptosis [65], manifesting metabolic abnormalities [66]. However, recent studies have demonstrated that the mitochondrial theory of aging appears to have been overestimated [67, 68], indicating no necessary connection between aging and mitochondria-derived ROS production [67]. In fact, cardiomyocytes appear to focus on injury tolerance and repair [69] instead of apoptosis and regeneration [70]. Interestingly, mild ROS are validated to be beneficial to the heart to some degree [71], such as ischemic preconditioning (IPC) [72] and the protective effects induced by physical exercise [73]. A probable mechanism might be the mitohormesis [74] and AMPK/Unc-51 like autophagy activating kinase1 (ULK1)-mediated pro-autophagy pathway [75]. Moderate ROS within mitochondria may develop an adaptive reaction, finally causing cellular stress resistance and OS inhibition. Mitohormesis, an inhibitory process for OS, is beneficial for extending lifespan induced by physical exercise [73, 76]. Despite the protective effect, excessive ROS might be harmful during ischemic-reperfusion injury (IRI) [77]. Long-term exposure to ROS is capable of promoting cardiac hypertrophy by inducing cardiomyocytes apoptosis, necrosis, and fibrosis [78, 79]. In conclusion, low dose of ROS contributes to health-promoting capability while higher dose and sustained stimulation of ROS may lead to OS [80]. Further studies on the impact of ROS are still needed.

## Protective factors for HF

### FGF21

FGF21, a novel member of fibroblast growth factors (FGFs), was first isolated from mouse embryos by Nishimura et al [81]. FGF21 is mainly expressed in the liver, pancreas, and adipose tissues [82, 83], partially expressed in the myocardium [84]. The endogenous receptors of FGF21 include FGF receptor 1 (FGFR1) and β-Klotho [85] that are also highly expressed in the myocardium [84]. Clinical studies have shown elevated levels of circulating FGF21 in patients with atherosclerosis or those at high risk of atherosclerosis [86]. The application of exogenous FGF21 is capable of significantly improving the lipid metabolism disorder in mice and reducing the area of atherosclerotic plaque [87]. Mice lacking FGF21 are more prone to hypercholesterolemia and atherosclerosis [88], suggesting the cardioprotective effect of elevated FGF21. In IRI models, FGF21 bound to cardiac receptors to activate the Akt1-glycogen synthase kinase-3β-caspase 3 (Akt1-GSK-3β-caspase 3) signal pathway [89], then phosphorylating phosphoinositide 3-kinase (PI3K), p85, Akt1 and BCL-2/BCL-XL-associated death promoter (BAD), consequently reducing the activity of caspase 3 and apoptosis of cardiomyocytes [90]. Mitochondrial uncoupling protein 3 (UCP3) exerts an anti-OS function by activating FGF21 under myocardial hypertrophy condition [91]. Similarly, genetic deletion of UCP3 exaggerates the expression of apoptotic signal, leading to HF [92]. Additionally, FGF21 is also capable of inhibiting the ROS production by activating superoxide dismutase 2 (SOD2) via extracellular signal-regulated kinases (EPKs) on the basis of sirtuin1 (SIRT1) overexpression [84, 93]. Correspondingly, patients with HF showed upregulation of UCP3 and SOD2 [26].

Autophagy deficiency is associated with modifiable factors of atherosclerosis [46], such as IR, dyslipidemia, and abdominal obesity [27, 94]. FGF21 is activated by activating transcription factor 4 (ATF4) induced by autophagy deficiency [95], protecting mice against diet-induced obesity [96] by enhancing the mitochondria oxidative efficiency [97], increasing fatty acid utilization, promoting lipid excretion [98], and lowering the level of blood glucose and triglycerides [99]. Accordingly, the deficiency of FGF21 enhanced myocardial lipid accumulation in mice [27]. As per a clinical study, advancements were preliminarily obtained in patients with obesity which ameliorates dyslipidemia by applying FGF21 analogues [100].

### MDPs

MDPs are a class of peptides encoded by mitochondrial deoxyribonucleic acid (DNA), mainly including humanin, mitochondrial open reading frame of the 12S rRNA-c (MOTS-c), and small humanin-like peptides (SHLPs) [101, 102]. As the first member of MDPs, humanin has been validated to induce positive metabolic activities, such as reduction in visceral fat and increase in glucose-stimulated insulin release [101]. MOTS-c and SHLPs further complement the role of MDPs in cell metabolism [103, 104]. Recently, the metabolic protection mechanism of MDPs in CVDs has been gradually recognized [105].

### Humanin and SHLPs

Humanin is a micropeptide encoded by the 16S ribosomal ribonucleic acid (RNA) gene of mitochondrial genome, discovered by Hashimoto Yuichi [106]. Humanin was initially thought to be a specific neuronal protective peptide for AD [107]. Recent studies demonstrated that humanin plays an essentially protective role in cardiac stress [108]. In IRI models, humanin protects left ventricular function [109] by promoting mitochondrial biogenesis [110] and the expression of endothelial nitric oxide synthase (eNOS) [111]. Additionally, [Gly14]-humanin (HNG) can reduce the risk of atherosclerosis by increasing cholesterol efflux and reducing the uptake the oxidized low-density lipoprotein (ox-LDL) by macrophage-derived foam cells [112]. Small humanin-like peptides (SHLPs) are also a class of polypeptides encoded in the mitochondrial 16S rRNA region. Six peptides (SHLP1~6) have been identified so far, each of which is 20–38-amino acids long [104]. SHLP2 exhibits a similar effect to HN in anti-apoptosis, insulin sensitization, and glucose homeostasis maintenance [104]. In addition, in-vitro studies have demonstrated that SHLP2 are capable of improving mitochondrial metabolism by increasing the oxygen consumption rate (OCR) and ATP generation [113]. SHLP2 reportedly activate the signal transducers and activators of transcription 3 (STAT3) pathway in a time-dependent manner; however, the specific mechanism remains still unclear [104].

### MOTS-c

MOTS-c is encoded by mitochondrial 12S rRNA [103] that is activated by metabolic stress signals and transferred to the nucleus, regulating adaptive nuclear gene expression [114]. The polymorphism of MOTS-c is related to longevity [28], playing a vital role in regulating obesity and diabetes [115]. MOTS-c is capable of reversing age-dependent and HFD-induced insulin resistance, preventing diet-induced obesity [116]. Mechanistically, MOTS-c regulates cell metabolism by inhibiting the folate cycle, new purine biosynthesis, and endogenous aminoimidazole-4-carboxamide ribonucleotide (AICAR) aggregation via the folate-AICAR-AMPK pathway [103]. In addition, MOTS-c also plays an important role in protecting vascular endothelial function [117] by inhibiting the activity of mitogen-activated protein kinases (MAPKs) and reducing the expression of inflammatory factors (TNF-α, IL-6, IL-1β) induced by nuclear factor kappa-light-chain-enhancer of activated B cells (NF-κB) [118]. Consequently, lower endogenous MOTS-c level is thought to be associated with impaired coronary endothelial function [119].

## Potential diagnostic and therapeutic targets for HF

### GDF15

GDF15, first identified as macrophage inhibitory cytokine 1 (MIC-1), belongs to the transforming growth factor (TGF)-β superfamily [120] that plays a significant role in regulating the inflammatory pathway, cell growth, cell repair, and apoptosis [121]. Similar to FGF21, GDF15 is also considered a marker of mitochondrial respiratory chain deficiency [122]. Activated GDF15 combines with glial cell-derived neurotrophic factor (GDNF) family receptor α-like (GFRAL) [123] to regulate appetite and energy metabolism by affecting mitochondrial biogenesis, calorie production, and fatty acid metabolism [124]. Preliminary studies have considered GDF15 as a biomarker and prognostic indicator for HF [125]. Lok et al. [126] first reported that GDF-15 has the potential to estimate the prognosis of possible therapeutic interventions, such as left ventricular assist device (LVAD) implantation. Similarly, another clinical study demonstrated that GDF15 and N-terminal pro-brain natriuretic peptide (NT-proBNP) are both core biomarkers for patients with HFrEF and patients with HFpEF [127, 128]. However, the expression of GDF15 appears to be related to a variety of pathological states, suggesting that GDF15 might act as a general stress factor [129].

### Adropin and irisin

Adropin is a novel membrane-bound protein containing 76 amino acids encoded by an energy homeostasis-related gene (ENHO) [130]. It is mainly expressed in the liver, brain, coronary arteries, vascular endothelium, and heart [131]. Adropin is capable of improving cardiac glucose metabolism in mice with HFD [132] and regulating pyruvate dehydrogenase in cardiomyocytes via the G protein-coupled receptor-MAPK-pyruvate dehydrogenase lipoamide kinase isozyme 4 (GPCR-MAPK-PDK4) signal pathway [133], suggesting an important role of adropin in cardiac substrate utilization. Furthermore, adropin upregulates the expression of eNOS and protects endothelial function [134] via the vascular endothelial growth factor receptor 2 (VEGFR2)-phosphatidylinositol 3-kinase-Akt and VEGFR2-ERK1/2 pathways [135]. Clinically, lower level of adropin is an independent risk factor for CVDs, and circulatory adropin level increases along with HF severity [136]. Collectively, these findings suggested that elevated adropin in HF patients improves cardiac function by regulating metabolism and protecting the vascular endothelium; further, adropin has the potential to be a serum biomarker for early diagnosis of CVDs.

Irisin is a polypeptide hormone that contains 112 amino acids and was discovered by Boström et al [137]. It is cleaved from Fibronectin type III domain-containing protein 5 (FNDC5) when activated by PGC-1α after exercise or stress [138]. Irisin is highly expressed in the myocardium, skeletal muscle, brain, and spinal cord [139]. Irisin is capable of converting white adipose tissue into brown adipose tissue by upregulating the expression of Ucp1 [137]. Elevated level of irisin has been recognized to highly correspond with many CVDs, suggesting poor prognosis [140]. Mechanistically, irisin protects against pressure overload-induced myocardial hypertrophy and ameliorates angiotensin II-induced cardiomyocyte apoptosis by activating AMPK-ULK1 signaling and inducing protective autophagy and autophagy flux [141]. Clinical studies have demonstrated that both adropin and irisin are related to HF severity [131] that might be an emerging marker of cardiac cachexia in HFrEF patients. Interestingly, a study has demonstrated that plasm level of irisin in HFpEF was obviously higher than patients with HFrEF. In addition, the negative relationship between irisin and total antioxidant capacity (TAC) was only observed in patients with HFpEF, suggesting a distinct mechanism of irisin secretion in the two HF subtypes [142].

### UPR^mt^

UPR^mt^ was first identified as a crucial regulatory pathway for mitochondrial protein homeostasis and quality control in Caenorhabditis elegans (C. elegans) [143]. Physiologically, nuclear-encoded proteins are transported to mitochondria by ribosomes [144] where they are properly folded and assembled [145]. During mitochondria stress, lower ATP or transmembrane potential in the cells slows down the process of precursor proteins entering the mitochondria, leading to a large number of misfolded proteins or protein precursors accumulating in the cytoplasm [146]. UPR^mt^ is subsequently activated by the mitochondrial proteasome to upregulate the expression of molecular chaperones, proteases, and antioxidant genes, restoring mitochondrial function [25]. Noticeably, UPR^mt^ is mainly regulated by activating transcription factor associated with stress-1 (ATFS-1) in models of worm and C. elegans [147]. ATFS-1 contains a mitochondrial targeting sequence (MTS) and a nuclear localization sequence (NLS), guaranteeing its regulation for communication from the mitochondria to the nucleus [148]. In the case of mitochondrial dysfunction, the mitochondrial importing ability decreases [149], leading to ATFS-1 accumulation in the cytoplasm. Subsequently, ATFS-1 enters the nucleus through NLS, activating nuclear transcription reaction [150] that weakens the expression of OXPHOS-related genes and strengthens the expression of molecular chaperone and proteasome-related genes to reduce ROS toxicity and increase mitochondrial importing ability, consequently reconstructing mitochondrial protein homeostasis [148]. Recent studies have demonstrated that mitochondrial stress induced by knockdown ETC subunits in C. elegans activates UPR^mt^ both in neurons and gut, improving health and prolonging life [20].

Recently, the metabolic regulation of UPR^mt^ has been gradually recognized [151]. Interestingly, the metabolic effects of UPR^mt^ on proliferating and post-mitotic cells are different. In proliferating cells, sustained UPR^mt^ promotes glycolysis while maintaining the mitochondrial function [152]. However, in mitotic or post-mitotic cells, such as muscle cells, UPR^mt^ inhibits the expression of tricarboxylic acid cycle and OXPHOS-related genes and reduces the metabolic load and cell damage caused by secondary product ROS while increasing the expression of glycolysis and amino acid decomposition genes to meet the cellular needs for ATP [153]. It seems to be a temporary way for muscle cells to respond to mitochondrial stress without permanently rewiring cell metabolism [154]. Notably, Smyrnias et al. [10] concluded that the pharmacodynamic enhancement of myocardial UPR^mt^ is capable of improving mitochondrial and systolic dysfunction, using vitro myocardial cell test, a mouse heart overload model, and plasma marker analysis of patients with aortic stenosis. They also demonstrated that UPR^mt^ activation is negatively correlated to lower plasma levels of high-sensitive cardiac troponin (hs-cNT) and N-terminal pro B type natriuretic peptide (NT-pro BNP) [155] in patients with aortic stenosis. UPR^mt^ is regulated by ATF5 in mammals [10], and NAD^+^ supplementation has the potential to improve UPR^mt^ activity [156]. Similar to ATFS-1, ATF5 is also a transcription factor containing the basic leucine zipper (bZip) domain [150]. Additionally, studies have confirmed that choline attenuates myocardial hypertrophy by modulating the expression of UPR^mt^ [157]. However, excessive prolongation or lack UPR^mt^ regulation might cause harm by contributing to the accumulation of defective mitochondria [158] and the formation of neurodegenerative phenotypes [159].

## Possibilities and challenges

**Figure 3:**
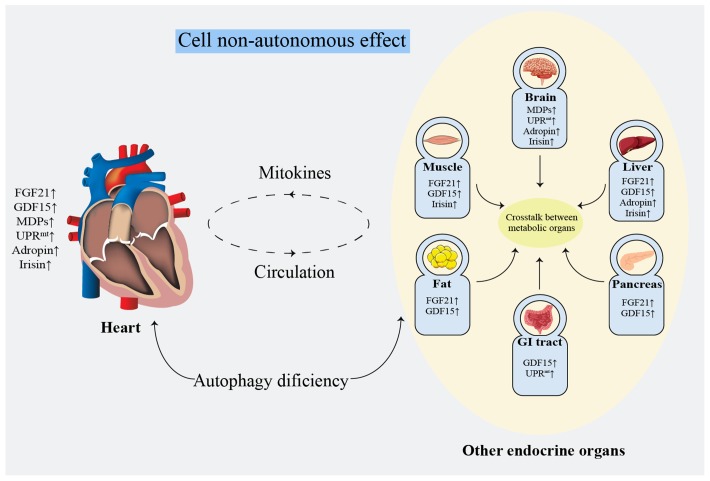
**Systematic metabolism regulated by cell non-autonomous effect.** FGF21: fibroblast growth factor 21; GDF15: growth differentiation factor 15; GI: gastrointestinal; MDPs: mitochondria-derived peptides; UPR^mt^: mitochondrial unfolded protein response.

Non-invasive evaluation of mitochondrial function remains unresolved [160]. Clinically, biomarkers with a high specificity and short-term sensitivity are urgently needed [32]. The lactate: pyruvate ratio [161] and oxidative damage markers [162] can be referenced to evaluate systemic mitochondrial function. Additionally, FGF21 [163] and GDF15 [122] have been validated as biomarkers for mitochondrial diseases in mouse models and patients. However, the specificity of these methods remains unsatisfactory [32]. Noticeably, it seems more meaningful to focus on the protective effects on the heart rather than distinguish the origins of mitokines. Mitokines secreted by other tissues reduce the HF risk by ameliorating IR and regulating glucose and lipid metabolism [96, 104, 124]. Damaged myocardial cells or endothelial cells simultaneously secret mitokines into the circulation, regulating lipid metabolism and protecting against oxidative stress or inflammatory injury by affecting the cell surface receptors, consequently improving atherosclerosis, protecting the ischemic myocardium and reducing IRI [90]. These findings suggest that mitokines protect against cardiac damage by systemic metabolic regulation effect (Figure 3). Mechanistically, the nucleus regulates mitochondrial metabolism through FGF21, GDF15, adropin, and irisin, while the mitochondria retrogradely regulates nuclear metabolism-related gene expression by MDPs and UPR^mt^ [164] that changes the traditional understanding of the mitochondria as terminal functional organelles that receive cell signals (Figure 2).

The existing literature shows differences in the research results for partial mitokines. Circulating levels of FGF21 have been reported to be positively correlated with age, causing premature aging and death in mice [165]. Similarly, it is suggested that the beneficial results of muscle mitochondrial stress might be independent of endogenous FGF21 activation [166]. Furthermore, several observations have indicated that the effect of GDF15 might highly depend on the state of the cell and its environment [167]. Despite the positive effects of mitokines [97, 98, 121, 129], the specific metabolic mechanism of mitokines requires greater clarification. In addition, obesity has been validated to be a FGF21 resistance state, and that further studies on addressing FGF21 resistance are needed [168]. Decreased activity of FGF21 has been observed in heart samples from obese rodents and white adipose tissue of human due to the decreased expression of beta-klotho [169, 170]. Although this conclusion was under challenge [171], the signal response of ERK1/2 phosphorylation was significantly weakened when using exogenous FGF21 to treat obese mice, along with the impaired induction of FGF21 target genes (cFos and EGR1) [168]. Objectively, the differences in the experimental data of animal models and human HF patients might be owing to the diversity in the species and dietary styles [172]. Furthermore, alterations in metabolism tend to occur in late stages in animal HF models. In addition, gross and micro differences in metabolic changes might be observed in the left ventricle [12]. Hence, drawing a general conclusion from a single point in time or from a single animal model needs careful considerations [173].

## Outlook

The importance of metabolic alterations in the myocardium for the subsequent development of HF has been previously highlighted [12]. The interactions between mitochondrial dysfunction and HF have been continuously examined [14, 32]. It is difficult to evaluate mitochondrial function noninvasively, achieve targeted drug delivery, and reduce drug toxicity [160, 174]. Noticeably, the emerging concept of mitokines might represent a novel prospect for HF therapy. It has great potential in the diagnosis and treatment of CVDs if these processes are well understood and the related genes and peptides are further identified. Currently, adropin and irisin have shown a correlation with HF severity and might be emerging markers of HF [131]. FGF21 and GDF15 have already been investigated in pre-clinical studies [128, 175], especially in the treatment of adrenergic nervous system (ANS) hyperactivity-induced HF [176]. It has been reported that up-regulation of GDF15 negatively regulated norepinephrine-induced myocardial hypertrophy by inhibiting epidermal growth factor receptor (EGFR) transactivation [177]. Similarly, FGF21 reduced angiotensin II (Ang II)-induced myocardial hypertrophy through SIRT1 [178]. Given the fact that the influential effects of physical exercise and Ang II type 1 (AT_1_) receptor antagonists on GDF15 and FGF21 [179, 180], whether GDF15 and FGF21 have the potential to evaluate the prognosis of patients with HF treated by angiotensin receptor-neprilysin inhibitor (ARNI) has not yet been determined. MDPs [105] and UPR^mt^ [157] are typical examples of mitochondrial reverse regulation of nuclear metabolic gene expression, providing a novel therapeutic approach. As previously mentioned, FGF21 [90], MOTS-c [103], and irisin [181] are regulated by AMPK; ATFs activates UPR^mt^ [10] and FGF21; ERK1/2 is activated by HNG, SHLPs [104], and FGF21 [90]. Furthermore, MOTS-c also reduces the myocardial immune response and protects endothelial function by inhibiting MAPKs [117, 118]. Hence, studying the metabolism-related signaling pathways and transcription factors can deepen our understanding of mitokines-mediated cardiac protection.

Considering the current progress with mitokines [165], we hope to establish a validated class of biomarkers and predictive algorithms that are capable of screening patients with risk factors of HF before clinical symptoms emerge, assisting subsequent treatment. We have only discussed a small fraction of the possibilities of mitokines as a therapeutic target for HF. For example, the secretion of partial mitokines is influenced by circadian and nutritional factors [182] that might lower its specificity. In addition, high-risk factors, such as diabetes, obesity, and liver diseases should be carefully evaluated. However, we still encourage clinicians to explore the possibilities in appropriate patient populations.

The communication among the adipose tissue, skeletal muscle, liver, heart, pancreas, intestine, and other major endocrine organs plays a crucial role in regulating energy metabolism [183, 184]. The effect of mitokines on cardiac and overall metabolic levels provides a novel hope for HF therapy (Figure3).

## References

[1] Yancy CW, Jessup M, Bozkurt B, Butler J, Casey DE Jr, Colvin MM, Drazner MH, Filippatos GS, Fonarow GC, Givertz MM, Hollenberg SM, Lindenfeld J, Masoudi FA, et al. 2017 ACC/AHA/HFSA Focused Update of the 2013 ACCF/AHA Guideline for the Management of Heart Failure: A Report of the American College of Cardiology/American Heart Association Task Force on Clinical Practice Guidelines and the Heart Failure Society of America. Circulation. 2017; 136:e137–61. 10.1161/CIR.0000000000000509 28455343

[2] Metra M, Teerlink JR. Heart failure. Lancet. 2017; 390:1981–95. 10.1016/S0140-6736(17)31071-1 28460827

[3] Lim GB. Acute coronary syndromes: silent myocardial infarction increases the risk of heart failure. Nat Rev Cardiol. 2018; 15:136. 10.1038/nrcardio.2018.4 29368702

[4] Lara KM, Levitan EB, Gutierrez OM, Shikany JM, Safford MM, Judd SE, Rosenson RS. Dietary Patterns and Incident Heart Failure in U.S. Adults Without Known Coronary Disease. J Am Coll Cardiol. 2019; 73:2036–45. 10.1016/j.jacc.2019.01.067 31023426 PMC6501554

[5] Brown DA, Perry JB, Allen ME, Sabbah HN, Stauffer BL, Shaikh SR, Cleland JG, Colucci WS, Butler J, Voors AA, Anker SD, Pitt B, Pieske B, et al. Expert consensus document: mitochondrial function as a therapeutic target in heart failure. Nat Rev Cardiol. 2017; 14:238–50. 10.1038/nrcardio.2016.203 28004807 PMC5350035

[6] Kaski JC, Gloekler S, Ferrari R, Fox K, Lévy BI, Komajda M, Vardas P, Camici PG. Role of ivabradine in management of stable angina in patients with different clinical profiles. Open Heart. 2018; 5:e000725. 10.1136/openhrt-2017-000725 29632676 PMC5888443

[7] Downey JM, Cohen MV. Why do we still not have cardioprotective drugs? Circ J. 2009; 73:1171–77. 10.1253/circj.CJ-09-0338 19506318

[8] Yau TM, Pagani FD, Mancini DM, Chang HL, Lala A, Woo YJ, Acker MA, Selzman CH, Soltesz EG, Kern JA, Maltais S, Charbonneau E, Pan S, et al, and Cardiothoracic Surgical Trials Network. Intramyocardial Injection of Mesenchymal Precursor Cells and Successful Temporary Weaning From Left Ventricular Assist Device Support in Patients With Advanced Heart Failure: A Randomized Clinical Trial. JAMA. 2019; 321:1176–86. 10.1001/jama.2019.2341 30912838 PMC6439694

[9] Curfman G. Stem Cell Therapy for Heart Failure: An Unfulfilled Promise? JAMA. 2019; 321:1186–87. 10.1001/jama.2019.2617 30912818

[10] Smyrnias I, Gray SP, Okonko DO, Sawyer G, Zoccarato A, Catibog N, López B, González A, Ravassa S, Díez J, Shah AM. Cardioprotective Effect of the Mitochondrial Unfolded Protein Response During Chronic Pressure Overload. J Am Coll Cardiol. 2019; 73:1795–806. 10.1016/j.jacc.2018.12.087 30975297 PMC6456800

[11] Neubauer S. The failing heart—an engine out of fuel. N Engl J Med. 2007; 356:1140–51. 10.1056/NEJMra063052 17360992

[12] Schulz TJ, Westermann D, Isken F, Voigt A, Laube B, Thierbach R, Kuhlow D, Zarse K, Schomburg L, Pfeiffer AF, Tschöpe C, Ristow M. Activation of mitochondrial energy metabolism protects against cardiac failure. Aging (Albany NY). 2010; 2:843–53. 10.18632/aging.100234 21084725 PMC3006026

[13] Münzel T, Camici GG, Maack C, Bonetti NR, Fuster V, Kovacic JC. Impact of Oxidative Stress on the Heart and Vasculature: Part 2 of a 3-Part Series. J Am Coll Cardiol. 2017; 70:212–29. 10.1016/j.jacc.2017.05.035 28683969 PMC5663297

[14] Bertero E, Maack C. Metabolic remodelling in heart failure. Nat Rev Cardiol. 2018; 15:457–70. 10.1038/s41569-018-0044-6 29915254

[15] Verwoerd WS. A new computational method to split large biochemical networks into coherent subnets. BMC Syst Biol. 2011; 5:25. 10.1186/1752-0509-5-25 21294924 PMC3045323

[16] Shi L, Tu BP. Acetyl-CoA and the regulation of metabolism: mechanisms and consequences. Curr Opin Cell Biol. 2015; 33:125–31. 10.1016/j.ceb.2015.02.003 25703630 PMC4380630

[17] Chandel NS. Evolution of Mitochondria as Signaling Organelles. Cell Metab. 2015; 22:204–06. 10.1016/j.cmet.2015.05.013 26073494

[18] Haynes CM, Fiorese CJ, Lin YF. Evaluating and responding to mitochondrial dysfunction: the mitochondrial unfolded-protein response and beyond. Trends Cell Biol. 2013; 23:311–18. 10.1016/j.tcb.2013.02.002 23489877 PMC3700555

[19] Mills E, O’Neill LA. Succinate: a metabolic signal in inflammation. Trends Cell Biol. 2014; 24:313–20. 10.1016/j.tcb.2013.11.008 24361092

[20] Sorrentino V, Menzies KJ, Auwerx J. Repairing Mitochondrial Dysfunction in Disease. Annu Rev Pharmacol Toxicol. 2018; 58:353–89. 10.1146/annurev-pharmtox-010716-104908 28961065

[21] Durieux J, Wolff S, Dillin A. The cell-non-autonomous nature of electron transport chain-mediated longevity. Cell. 2011; 144:79–91. 10.1016/j.cell.2010.12.016 21215371 PMC3062502

[22] Kim KH, Jeong YT, Oh H, Kim SH, Cho JM, Kim YN, Kim SS, Kim DH, Hur KY, Kim HK, Ko T, Han J, Kim HL, et al. Autophagy deficiency leads to protection from obesity and insulin resistance by inducing Fgf21 as a mitokine. Nat Med. 2013; 19:83–92. 10.1038/nm.3014 23202295

[23] Kim KH, Lee MS. Autophagy as a crosstalk mediator of metabolic organs in regulation of energy metabolism. Rev Endocr Metab Disord. 2014; 15:11–20. 10.1007/s11154-013-9272-6 24085381

[24] Jensen MB, Jasper H. Mitochondrial proteostasis in the control of aging and longevity. Cell Metab. 2014; 20:214–25. 10.1016/j.cmet.2014.05.006 24930971 PMC4274350

[25] Münch C, Harper JW. Mitochondrial unfolded protein response controls matrix pre-RNA processing and translation. Nature. 2016; 534:710–13. 10.1038/nature18302 27350246 PMC4939261

[26] Foote K, Bennett MR. Molecular insights into vascular aging. Aging (Albany NY). 2018; 10:3647–49. 10.18632/aging.101697 30521484 PMC6326650

[27] Crupi AN, Nunnelee JS, Taylor DJ, Thomas A, Vit JP, Riera CE, Gottlieb RA, Goodridge HS. Oxidative muscles have better mitochondrial homeostasis than glycolytic muscles throughout life and maintain mitochondrial function during aging. Aging (Albany NY). 2018; 10:3327–52. 10.18632/aging.101643 30449736 PMC6286850

[28] Fuku N, Pareja-Galeano H, Zempo H, Alis R, Arai Y, Lucia A, Hirose N. The mitochondrial-derived peptide MOTS-c: a player in exceptional longevity? Aging Cell. 2015; 14:921–23. 10.1111/acel.12389 26289118 PMC4693465

[29] Schulze PC, Drosatos K, Goldberg IJ. Lipid Use and Misuse by the Heart. Circ Res. 2016; 118:1736–51. 10.1161/CIRCRESAHA.116.306842 27230639 PMC5340419

[30] Amgalan D, Chen Y, Kitsis RN. Death Receptor Signaling in the Heart: Cell Survival, Apoptosis, and Necroptosis. Circulation. 2017; 136:743–46. 10.1161/CIRCULATIONAHA.117.029566 28827219 PMC5657500

[31] Ago T, Matsushima S, Kuroda J, Zablocki D, Kitazono T, Sadoshima J. The NADPH oxidase Nox4 and aging in the heart. Aging (Albany NY). 2010; 2:1012–16. 10.18632/aging.100261 21212466 PMC3034169

[32] Murphy MP, Hartley RC. Mitochondria as a therapeutic target for common pathologies. Nat Rev Drug Discov. 2018; 17:865–86. 10.1038/nrd.2018.174 30393373

[33] Dimroth P, Kaim G, Matthey U. Crucial role of the membrane potential for ATP synthesis by F(1)F(o) ATP synthases. J Exp Biol. 2000; 203:51–59. 10600673 10.1242/jeb.203.1.51

[34] Abozguia K, Shivu GN, Ahmed I, Phan TT, Frenneaux MP. The heart metabolism: pathophysiological aspects in ischaemia and heart failure. Curr Pharm Des. 2009; 15:827–35. 10.2174/138161209787582101 19275646

[35] Randle PJ, Garland PB, Hales CN, Newsholme EA. The glucose fatty-acid cycle. Its role in insulin sensitivity and the metabolic disturbances of diabetes mellitus. Lancet. 1963; 1:785–89. 10.1016/S0140-6736(63)91500-9 13990765

[36] Jeon SM. Regulation and function of AMPK in physiology and diseases. Exp Mol Med. 2016; 48:e245. 10.1038/emm.2016.81 27416781 PMC4973318

[37] Hardie DG, Ross FA, Hawley SA. AMPK: a nutrient and energy sensor that maintains energy homeostasis. Nat Rev Mol Cell Biol. 2012; 13:251–62. 10.1038/nrm3311 22436748 PMC5726489

[38] Lehman JJ, Barger PM, Kovacs A, Saffitz JE, Medeiros DM, Kelly DP. Peroxisome proliferator-activated receptor gamma coactivator-1 promotes cardiac mitochondrial biogenesis. J Clin Invest. 2000; 106:847–56. 10.1172/JCI10268 11018072 PMC517815

[39] Schlattner U, Tokarska-Schlattner M, Wallimann T. Mitochondrial creatine kinase in human health and disease. Biochim Biophys Acta. 2006; 1762:164–80. 10.1016/j.bbadis.2005.09.004 16236486

[40] Balaban RS, Kantor HL, Katz LA, Briggs RW. Relation between work and phosphate metabolite in the in vivo paced mammalian heart. Science. 1986; 232:1121–23. 10.1126/science.3704638 3704638

[41] Bedi KC Jr, Snyder NW, Brandimarto J, Aziz M, Mesaros C, Worth AJ, Wang LL, Javaheri A, Blair IA, Margulies KB, Rame JE. Evidence for Intramyocardial Disruption of Lipid Metabolism and Increased Myocardial Ketone Utilization in Advanced Human Heart Failure. Circulation. 2016; 133:706–16. 10.1161/CIRCULATIONAHA.115.017545 26819374 PMC4779339

[42] Chandler MP, Kerner J, Huang H, Vazquez E, Reszko A, Martini WZ, Hoppel CL, Imai M, Rastogi S, Sabbah HN, Stanley WC. Moderate severity heart failure does not involve a downregulation of myocardial fatty acid oxidation. Am J Physiol Heart Circ Physiol. 2004; 287:H1538–43. 10.1152/ajpheart.00281.2004 15191896

[43] Nascimben L, Ingwall JS, Lorell BH, Pinz I, Schultz V, Tornheim K, Tian R. Mechanisms for increased glycolysis in the hypertrophied rat heart. Hypertension. 2004; 44:662–67. 10.1161/01.HYP.0000144292.69599.0c 15466668

[44] Aubert G, Martin OJ, Horton JL, Lai L, Vega RB, Leone TC, Koves T, Gardell SJ, Krüger M, Hoppel CL, Lewandowski ED, Crawford PA, Muoio DM, Kelly DP. The Failing Heart Relies on Ketone Bodies as a Fuel. Circulation. 2016; 133:698–705. 10.1161/CIRCULATIONAHA.115.017355 26819376 PMC4766035

[45] Ferrannini E, Mark M, Mayoux E. CV Protection in the EMPA-REG OUTCOME Trial: A “Thrifty Substrate” Hypothesis. Diabetes Care. 2016; 39:1108–14. 10.2337/dc16-0330 27289126

[46] Di Pino A, DeFronzo RA. Insulin Resistance and Atherosclerosis: Implications for Insulin Sensitizing Agents. Endocr Rev. 2019; [Epub ahead of print]. 10.1210/er.2018-00141 31050706 PMC7445419

[47] Riehle C, Abel ED. Insulin Signaling and Heart Failure. Circ Res. 2016; 118:1151–69. 10.1161/CIRCRESAHA.116.306206 27034277 PMC4833475

[48] Ceylan-Isik AF, Kandadi MR, Xu X, Hua Y, Chicco AJ, Ren J, Nair S. Apelin administration ameliorates high fat diet-induced cardiac hypertrophy and contractile dysfunction. J Mol Cell Cardiol. 2013; 63:4–13. 10.1016/j.yjmcc.2013.07.002 23859766

[49] Lavie CJ, Alpert MA, Arena R, Mehra MR, Milani RV, Ventura HO. Impact of obesity and the obesity paradox on prevalence and prognosis in heart failure. JACC Heart Fail. 2013; 1:93–102. 10.1016/j.jchf.2013.01.006 24621833

[50] Kenchaiah S, Pocock SJ, Wang D, Finn PV, Zornoff LA, Skali H, Pfeffer MA, Yusuf S, Swedberg K, Michelson EL, Granger CB, McMurray JJ, Solomon SD, and CHARM Investigators. Body mass index and prognosis in patients with chronic heart failure: insights from the Candesartan in Heart failure: Assessment of Reduction in Mortality and morbidity (CHARM) program. Circulation. 2007; 116:627–36. 10.1161/CIRCULATIONAHA.106.679779 17638930

[51] Cao C, Wang R, Wang J, Bunjhoo H, Xu Y, Xiong W. Body mass index and mortality in chronic obstructive pulmonary disease: a meta-analysis. PLoS One. 2012; 7:e43892. 10.1371/journal.pone.0043892 22937118 PMC3427325

[52] Veronese N, Cereda E, Solmi M, Fowler SA, Manzato E, Maggi S, Manu P, Abe E, Hayashi K, Allard JP, Arendt BM, Beck A, Chan M, et al. Inverse relationship between body mass index and mortality in older nursing home residents: a meta-analysis of 19,538 elderly subjects. Obes Rev. 2015; 16:1001–15. 10.1111/obr.12309 26252230

[53] Schmidt DS, Salahudeen AK. Obesity-survival paradox-still a controversy? Semin Dial. 2007; 20:486–92. 10.1111/j.1525-139X.2007.00349.x 17991192

[54] Kalantar-Zadeh K, Block G, Horwich T, Fonarow GC. Reverse epidemiology of conventional cardiovascular risk factors in patients with chronic heart failure. J Am Coll Cardiol. 2004; 43:1439–44. 10.1016/j.jacc.2003.11.039 15093881

[55] Sharma A, Lavie CJ, Borer JS, Vallakati A, Goel S, Lopez-Jimenez F, Arbab-Zadeh A, Mukherjee D, Lazar JM. Meta-analysis of the relation of body mass index to all-cause and cardiovascular mortality and hospitalization in patients with chronic heart failure. Am J Cardiol. 2015; 115:1428–34. 10.1016/j.amjcard.2015.02.024 25772740

[56] Pittman JG, Cohen P. The Pathogenesis of Cardiac Cachexia. N Engl J Med. 1964; 271:453–60. 10.1056/NEJM196408272710908 14171818

[57] Anker SD, Ponikowski PP, Clark AL, Leyva F, Rauchhaus M, Kemp M, Teixeira MM, Hellewell PG, Hooper J, Poole-Wilson PA, Coats AJ. Cytokines and neurohormones relating to body composition alterations in the wasting syndrome of chronic heart failure. Eur Heart J. 1999; 20:683–93. 10.1053/euhj.1998.1446 10208789

[58] Sandek A, Bauditz J, Swidsinski A, Buhner S, Weber-Eibel J, von Haehling S, Schroedl W, Karhausen T, Doehner W, Rauchhaus M, Poole-Wilson P, Volk HD, Lochs H, Anker SD. Altered intestinal function in patients with chronic heart failure. J Am Coll Cardiol. 2007; 50:1561–69. 10.1016/j.jacc.2007.07.016 17936155

[59] Poehlman ET, Scheffers J, Gottlieb SS, Fisher ML, Vaitekevicius P. Increased resting metabolic rate in patients with congestive heart failure. Ann Intern Med. 1994; 121:860–62. 10.7326/0003-4819-121-11-199412010-00006 7772113

[60] Vest AR, Chan M, Deswal A, Givertz MM, Lekavich C, Lennie T, Litwin SE, Parsly L, Rodgers JE, Rich MW, Schulze PC, Slader A, Desai A. Nutrition, Obesity, and Cachexia in Patients With Heart Failure: A Consensus Statement from the Heart Failure Society of America Scientific Statements Committee. J Card Fail. 2019; 25:380–400. 10.1016/j.cardfail.2019.03.007 30877038

[61] Coats AJ. Research on cachexia, sarcopenia and skeletal muscle in cardiology. J Cachexia Sarcopenia Muscle. 2012; 3:219–23. 10.1007/s13539-012-0090-6 23160775 PMC3505572

[62] Di Angelantonio E, Bhupathiraju SN, Wormser D, Gao P, Kaptoge S, Berrington de Gonzalez A, Cairns BJ, Huxley R, Jackson CL, Joshy G, Lewington S, Manson JE, Murphy N, et al, and Global BMI Mortality Collaboration. Body-mass index and all-cause mortality: individual-participant-data meta-analysis of 239 prospective studies in four continents. Lancet. 2016; 388:776–86. 10.1016/S0140-6736(16)30175-1 27423262 PMC4995441

[63] Tadic M, Cuspidi C. Obesity and heart failure with preserved ejection fraction: a paradox or something else? Heart Fail Rev. 2019; 24:379–85. 10.1007/s10741-018-09766-x 30610456

[64] Mohammed SF, Borlaug BA, Roger VL, Mirzoyev SA, Rodeheffer RJ, Chirinos JA, Redfield MM. Comorbidity and ventricular and vascular structure and function in heart failure with preserved ejection fraction: a community-based study. Circ Heart Fail. 2012; 5:710–19. 10.1161/CIRCHEARTFAILURE.112.968594 23076838 PMC3767407

[65] Matés JM, Segura JA, Alonso FJ, Márquez J. Intracellular redox status and oxidative stress: implications for cell proliferation, apoptosis, and carcinogenesis. Arch Toxicol. 2008; 82:273–99. 10.1007/s00204-008-0304-z 18443763

[66] Emelyanova L, Preston C, Gupta A, Viqar M, Negmadjanov U, Edwards S, Kraft K, Devana K, Holmuhamedov E, O’Hair D, Tajik AJ, Jahangir A. Effect of Aging on Mitochondrial Energetics in the Human Atria. J Gerontol A Biol Sci Med Sci. 2018; 73:608–16. 10.1093/gerona/glx160 28958065 PMC5905598

[67] López-Otín C, Blasco MA, Partridge L, Serrano M, Kroemer G. The hallmarks of aging. Cell. 2013; 153:1194–217. 10.1016/j.cell.2013.05.039 23746838 PMC3836174

[68] Tepp K, Timohhina N, Puurand M, Klepinin A, Chekulayev V, Shevchuk I, Kaambre T. Bioenergetics of the aging heart and skeletal muscles: modern concepts and controversies. Ageing Res Rev. 2016; 28:1–14. 10.1016/j.arr.2016.04.001 27063513

[69] Pohjoismäki JL, Goffart S. The role of mitochondria in cardiac development and protection. Free Radic Biol Med. 2017; 106:345–54. 10.1016/j.freeradbiomed.2017.02.032 28216385

[70] Chiong M, Wang ZV, Pedrozo Z, Cao DJ, Troncoso R, Ibacache M, Criollo A, Nemchenko A, Hill JA, Lavandero S. Cardiomyocyte death: mechanisms and translational implications. Cell Death Dis. 2011; 2:e244. 10.1038/cddis.2011.130 22190003 PMC3252742

[71] Kornfeld OS, Hwang S, Disatnik MH, Chen CH, Qvit N, Mochly-Rosen D. Mitochondrial reactive oxygen species at the heart of the matter: new therapeutic approaches for cardiovascular diseases. Circ Res. 2015; 116:1783–99. 10.1161/CIRCRESAHA.116.305432 25999419 PMC4443500

[72] Cadenas S. ROS and redox signaling in myocardial ischemia-reperfusion injury and cardioprotection. Free Radic Biol Med. 2018; 117:76–89. 10.1016/j.freeradbiomed.2018.01.024 29373843

[73] Merry TL, Ristow M. Mitohormesis in exercise training. Free Radic Biol Med. 2016; 98:123–30. 10.1016/j.freeradbiomed.2015.11.032 26654757

[74] Cox CS, McKay SE, Holmbeck MA, Christian BE, Scortea AC, Tsay AJ, Newman LE, Shadel GS. Mitohormesis in Mice via Sustained Basal Activation of Mitochondrial and Antioxidant Signaling. Cell Metab. 2018; 28:776–86.e5. 10.1016/j.cmet.2018.07.011 30122556 PMC6221994

[75] Marin JJ, Lozano E, Perez MJ. Lack of mitochondrial DNA impairs chemical hypoxia-induced autophagy in liver tumor cells through ROS-AMPK-ULK1 signaling dysregulation independently of HIF-1α. Free Radic Biol Med. 2016; 101:71–84. 10.1016/j.freeradbiomed.2016.09.025 27687210

[76] Ristow M, Zarse K. How increased oxidative stress promotes longevity and metabolic health: the concept of mitochondrial hormesis (mitohormesis). Exp Gerontol. 2010; 45:410–18. 10.1016/j.exger.2010.03.014 20350594

[77] Zhang Y, Ren J. Targeting autophagy for the therapeutic application of histone deacetylase inhibitors in ischemia/reperfusion heart injury. Circulation. 2014; 129:1088–91. 10.1161/CIRCULATIONAHA.113.008115 24396040

[78] Shirakabe A, Ikeda Y, Sciarretta S, Zablocki DK, Sadoshima J. Aging and Autophagy in the Heart. Circ Res. 2016; 118:1563–76. 10.1161/CIRCRESAHA.116.307474 27174950 PMC4869999

[79] Chen YR, Zweier JL. Cardiac mitochondria and reactive oxygen species generation. Circ Res. 2014; 114:524–37. 10.1161/CIRCRESAHA.114.300559 24481843 PMC4118662

[80] Ristow M. Unraveling the truth about antioxidants: mitohormesis explains ROS-induced health benefits. Nat Med. 2014; 20:709–11. 10.1038/nm.3624 24999941

[81] Nishimura T, Nakatake Y, Konishi M, Itoh N. Identification of a novel FGF, FGF-21, preferentially expressed in the liver. Biochim Biophys Acta. 2000; 1492:203–06. 10.1016/S0167-4781(00)00067-1 10858549

[82] Izumiya Y, Bina HA, Ouchi N, Akasaki Y, Kharitonenkov A, Walsh K. FGF21 is an Akt-regulated myokine. FEBS Lett. 2008; 582:3805–10. 10.1016/j.febslet.2008.10.021 18948104 PMC2604129

[83] Zhang X, Yeung DC, Karpisek M, Stejskal D, Zhou ZG, Liu F, Wong RL, Chow WS, Tso AW, Lam KS, Xu A. Serum FGF21 levels are increased in obesity and are independently associated with the metabolic syndrome in humans. Diabetes. 2008; 57:1246–53. 10.2337/db07-1476 18252893

[84] Planavila A, Redondo I, Hondares E, Vinciguerra M, Munts C, Iglesias R, Gabrielli LA, Sitges M, Giralt M, van Bilsen M, Villarroya F. Fibroblast growth factor 21 protects against cardiac hypertrophy in mice. Nat Commun. 2013; 4:2019. 10.1038/ncomms3019 23771152

[85] BonDurant LD, Potthoff MJ. Fibroblast Growth Factor 21: A Versatile Regulator of Metabolic Homeostasis. Annu Rev Nutr. 2018; 38:173–96. 10.1146/annurev-nutr-071816-064800 29727594 PMC6964258

[86] Chow WS, Xu A, Woo YC, Tso AW, Cheung SC, Fong CH, Tse HF, Chau MT, Cheung BM, Lam KS. Serum fibroblast growth factor-21 levels are associated with carotid atherosclerosis independent of established cardiovascular risk factors. Arterioscler Thromb Vasc Biol. 2013; 33:2454–59. 10.1161/ATVBAHA.113.301599 23887638

[87] Wu X, Lü Y, Fu K, Wang S, Zhao D, Peng H, Fan Q, Lü Y, Xin M, Liu J. [Impact of exogenous fibroblast growth factor 21 on atherosclerosis in apolipoprotein E deficient mice]. Zhonghua Xin Xue Guan Bing Za Zhi. 2014; 42:126–31. 24735623

[88] Lin Z, Pan X, Wu F, Ye D, Zhang Y, Wang Y, Jin L, Lian Q, Huang Y, Ding H, Triggle C, Wang K, Li X, Xu A. Fibroblast growth factor 21 prevents atherosclerosis by suppression of hepatic sterol regulatory element-binding protein-2 and induction of adiponectin in mice. Circulation. 2015; 131:1861–71. 10.1161/CIRCULATIONAHA.115.015308 25794851 PMC4444420

[89] Cong WT, Ling J, Tian HS, Ling R, Wang Y, Huang BB, Zhao T, Duan YM, Jin LT, Li XK. Proteomic study on the protective mechanism of fibroblast growth factor 21 to ischemia-reperfusion injury. Can J Physiol Pharmacol. 2013; 91:973–84. 10.1139/cjpp-2012-0441 24117266

[90] Patel V, Adya R, Chen J, Ramanjaneya M, Bari MF, Bhudia SK, Hillhouse EW, Tan BK, Randeva HS. Novel insights into the cardio-protective effects of FGF21 in lean and obese rat hearts. PLoS One. 2014; 9:e87102. 10.1371/journal.pone.0087102 24498293 PMC3911936

[91] Mancini A, Vergani E, Bruno C, Olivieri G, Di Segni C, Silvestrini A, Venuti A, Favuzzi A, Meucci E. Oxidative stress as a possible mechanism underlying multi-hormonal deficiency in chronic heart failure. Eur Rev Med Pharmacol Sci. 2018; 22:3936–61. 29949170 10.26355/eurrev_201806_15279

[92] Perrino C, Schiattarella GG, Sannino A, Pironti G, Petretta MP, Cannavo A, Gargiulo G, Ilardi F, Magliulo F, Franzone A, Carotenuto G, Serino F, Altobelli GG, et al. Genetic deletion of uncoupling protein 3 exaggerates apoptotic cell death in the ischemic heart leading to heart failure. J Am Heart Assoc. 2013; 2:e000086. 10.1161/JAHA.113.000086 23688674 PMC3698767

[93] Lai L, Yan L, Gao S, Hu CL, Ge H, Davidow A, Park M, Bravo C, Iwatsubo K, Ishikawa Y, Auwerx J, Sinclair DA, Vatner SF, Vatner DE. Type 5 adenylyl cyclase increases oxidative stress by transcriptional regulation of manganese superoxide dismutase via the SIRT1/FoxO3a pathway. Circulation. 2013; 127:1692–701. 10.1161/CIRCULATIONAHA.112.001212 23536361 PMC3980473

[94] Mishra M, Muthuramu I, De Geest B. HDL dysfunction, function, and heart failure. Aging (Albany NY). 2019; 11:293–94. 10.18632/aging.101775 30654330 PMC6366992

[95] Dogan SA, Pujol C, Maiti P, Kukat A, Wang S, Hermans S, Senft K, Wibom R, Rugarli EI, Trifunovic A. Tissue-specific loss of DARS2 activates stress responses independently of respiratory chain deficiency in the heart. Cell Metab. 2014; 19:458–69. 10.1016/j.cmet.2014.02.004 24606902

[96] Xu X, Krumm C, So JS, Bare CJ, Holman C, Gromada J, Cohen DE, Lee AH. Preemptive Activation of the Integrated Stress Response Protects Mice From Diet-Induced Obesity and Insulin Resistance by Fibroblast Growth Factor 21 Induction. Hepatology. 2018; 68:2167–81. 10.1002/hep.30060 29698569 PMC6203669

[97] Chau MD, Gao J, Yang Q, Wu Z, Gromada J. Fibroblast growth factor 21 regulates energy metabolism by activating the AMPK-SIRT1-PGC-1alpha pathway. Proc Natl Acad Sci USA. 2010; 107:12553–58. 10.1073/pnas.1006962107 20616029 PMC2906565

[98] Coskun T, Bina HA, Schneider MA, Dunbar JD, Hu CC, Chen Y, Moller DE, Kharitonenkov A. Fibroblast growth factor 21 corrects obesity in mice. Endocrinology. 2008; 149:6018–27. 10.1210/en.2008-0816 18687777

[99] Kharitonenkov A, Shiyanova TL, Koester A, Ford AM, Micanovic R, Galbreath EJ, Sandusky GE, Hammond LJ, Moyers JS, Owens RA, Gromada J, Brozinick JT, Hawkins ED, et al. FGF-21 as a novel metabolic regulator. J Clin Invest. 2005; 115:1627–35. 10.1172/JCI23606 15902306 PMC1088017

[100] Gaich G, Chien JY, Fu H, Glass LC, Deeg MA, Holland WL, Kharitonenkov A, Bumol T, Schilske HK, Moller DE. The effects of LY2405319, an FGF21 analog, in obese human subjects with type 2 diabetes. Cell Metab. 2013; 18:333–40. 10.1016/j.cmet.2013.08.005 24011069

[101] Kim SJ, Xiao J, Wan J, Cohen P, Yen K. Mitochondrially derived peptides as novel regulators of metabolism. J Physiol. 2017; 595:6613–21. 10.1113/JP274472 28574175 PMC5663826

[102] Gong Z, Su K, Cui L, Tas E, Zhang T, Dong HH, Yakar S, Muzumdar RH. Central effects of humanin on hepatic triglyceride secretion. Am J Physiol Endocrinol Metab. 2015; 309:E283–92. 10.1152/ajpendo.00043.2015 26058861 PMC4525112

[103] Lee C, Zeng J, Drew BG, Sallam T, Martin-Montalvo A, Wan J, Kim SJ, Mehta H, Hevener AL, de Cabo R, Cohen P. The mitochondrial-derived peptide MOTS-c promotes metabolic homeostasis and reduces obesity and insulin resistance. Cell Metab. 2015; 21:443–54. 10.1016/j.cmet.2015.02.009 25738459 PMC4350682

[104] Cobb LJ, Lee C, Xiao J, Yen K, Wong RG, Nakamura HK, Mehta HH, Gao Q, Ashur C, Huffman DM, Wan J, Muzumdar R, Barzilai N, Cohen P. Naturally occurring mitochondrial-derived peptides are age-dependent regulators of apoptosis, insulin sensitivity, and inflammatory markers. Aging (Albany NY). 2016; 8:796–809. 10.18632/aging.100943 27070352 PMC4925829

[105] Yang Y, Gao H, Zhou H, Liu Q, Qi Z, Zhang Y, Zhang J. The role of mitochondria-derived peptides in cardiovascular disease: recent updates. Biomed Pharmacother. 2019; 117:109075. 10.1016/j.biopha.2019.109075 31185388

[106] Hashimoto Y, Niikura T, Tajima H, Yasukawa T, Sudo H, Ito Y, Kita Y, Kawasumi M, Kouyama K, Doyu M, Sobue G, Koide T, Tsuji S, et al. A rescue factor abolishing neuronal cell death by a wide spectrum of familial Alzheimer’s disease genes and Abeta. Proc Natl Acad Sci USA. 2001; 98:6336–41. 10.1073/pnas.101133498 11371646 PMC33469

[107] Matsuoka M. Protective effects of Humanin and calmodulin-like skin protein in Alzheimer’s disease and broad range of abnormalities. Mol Neurobiol. 2015; 51:1232–39. 10.1007/s12035-014-8799-1 24969584

[108] Widmer RJ, Flammer AJ, Herrmann J, Rodriguez-Porcel M, Wan J, Cohen P, Lerman LO, Lerman A. Circulating humanin levels are associated with preserved coronary endothelial function. Am J Physiol Heart Circ Physiol. 2013; 304:H393–97. 10.1152/ajpheart.00765.2012 23220334 PMC3774506

[109] Qin Q, Mehta H, Yen K, Navarrete G, Brandhorst S, Wan J, Delrio S, Zhang X, Lerman LO, Cohen P, Lerman A. Chronic treatment with the mitochondrial peptide humanin prevents age-related myocardial fibrosis in mice. Am J Physiol Heart Circ Physiol. 2018; 315:H1127–36. 10.1152/ajpheart.00685.2017 30004252 PMC6415743

[110] Qin Q, Jin J, He F, Zheng Y, Li T, Zhang Y, He J. Humanin promotes mitochondrial biogenesis in pancreatic MIN6 β-cells. Biochem Biophys Res Commun. 2018; 497:292–97. 10.1016/j.bbrc.2018.02.071 29432738

[111] Lee C, Yen K, Cohen P. Humanin: a harbinger of mitochondrial-derived peptides? Trends Endocrinol Metab. 2013; 24:222–28. 10.1016/j.tem.2013.01.005 23402768 PMC3641182

[112] Zhu WW, Wang SR, Liu ZH, Cao YJ, Wang F, Wang J, Liu CF, Xie Y, Xie Y, Zhang YL. Gly[14]-humanin inhibits ox-LDL uptake and stimulates cholesterol efflux in macrophage-derived foam cells. Biochem Biophys Res Commun. 2017; 482:93–99. 10.1016/j.bbrc.2016.10.138 27815075

[113] Mehta HH, Xiao J, Ramirez R, Miller B, Kim SJ, Cohen P, Yen K. Metabolomic profile of diet-induced obesity mice in response to humanin and small humanin-like peptide 2 treatment. Metabolomics. 2019; 15:88. 10.1007/s11306-019-1549-7 31172328 PMC6554247

[114] Kim KH, Son JM, Benayoun BA, Lee C. The Mitochondrial-Encoded Peptide MOTS-c Translocates to the Nucleus to Regulate Nuclear Gene Expression in Response to Metabolic Stress. Cell Metab. 2018; 28:516–24.e7. 10.1016/j.cmet.2018.06.008 29983246 PMC6185997

[115] Lee C, Kim KH, Cohen P. MOTS-c: A novel mitochondrial-derived peptide regulating muscle and fat metabolism. Free Radic Biol Med. 2016; 100:182–87. 10.1016/j.freeradbiomed.2016.05.015 27216708 PMC5116416

[116] Li Q, Lu H, Hu G, Ye Z, Zhai D, Yan Z, Wang L, Xiang A, Lu Z. Earlier changes in mice after D-galactose treatment were improved by mitochondria derived small peptide MOTS-c. Biochem Biophys Res Commun. 2019; 513:439–45. 10.1016/j.bbrc.2019.03.194 30967270

[117] Qin Q, Delrio S, Wan J, Jay Widmer R, Cohen P, Lerman LO, Lerman A. Downregulation of circulating MOTS-c levels in patients with coronary endothelial dysfunction. Int J Cardiol. 2018; 254:23–27. 10.1016/j.ijcard.2017.12.001 29242099

[118] Zhai D, Ye Z, Jiang Y, Xu C, Ruan B, Yang Y, Lei X, Xiang A, Lu H, Zhu Z, Yan Z, Wei D, Li Q, et al. MOTS-c peptide increases survival and decreases bacterial load in mice infected with MRSA. Mol Immunol. 2017; 92:151–60. 10.1016/j.molimm.2017.10.017 29096170

[119] Maruhashi T, Kihara Y, Higashi Y. Assessment of endothelium-independent vasodilation: from methodology to clinical perspectives. J Hypertens. 2018; 36:1460–67. 10.1097/HJH.0000000000001750 29664811

[120] Bootcov MR, Bauskin AR, Valenzuela SM, Moore AG, Bansal M, He XY, Zhang HP, Donnellan M, Mahler S, Pryor K, Walsh BJ, Nicholson RC, Fairlie WD, et al. MIC-1, a novel macrophage inhibitory cytokine, is a divergent member of the TGF-beta superfamily. Proc Natl Acad Sci USA. 1997; 94:11514–19. 10.1073/pnas.94.21.11514 9326641 PMC23523

[121] Wollert KC, Kempf T, Lagerqvist B, Lindahl B, Olofsson S, Allhoff T, Peter T, Siegbahn A, Venge P, Drexler H, Wallentin L. Growth differentiation factor 15 for risk stratification and selection of an invasive treatment strategy in non ST-elevation acute coronary syndrome. Circulation. 2007; 116:1540–48. 10.1161/CIRCULATIONAHA.107.697714 17848615

[122] Yatsuga S, Fujita Y, Ishii A, Fukumoto Y, Arahata H, Kakuma T, Kojima T, Ito M, Tanaka M, Saiki R, Koga Y. Growth differentiation factor 15 as a useful biomarker for mitochondrial disorders. Ann Neurol. 2015; 78:814–23. 10.1002/ana.24506 26463265 PMC5057301

[123] Emmerson PJ, Wang F, Du Y, Liu Q, Pickard RT, Gonciarz MD, Coskun T, Hamang MJ, Sindelar DK, Ballman KK, Foltz LA, Muppidi A, Alsina-Fernandez J, et al. The metabolic effects of GDF15 are mediated by the orphan receptor GFRAL. Nat Med. 2017; 23:1215–19. 10.1038/nm.4393 28846098

[124] Fujita Y, Taniguchi Y, Shinkai S, Tanaka M, Ito M. Secreted growth differentiation factor 15 as a potential biomarker for mitochondrial dysfunctions in aging and age-related disorders. Geriatr Gerontol Int. 2016 (Suppl 1); 16:17–29. 10.1111/ggi.12724 27018280

[125] Wollert KC, Kempf T. Growth differentiation factor 15 in heart failure: an update. Curr Heart Fail Rep. 2012; 9:337–45. 10.1007/s11897-012-0113-9 22961192

[126] Lok SI, Winkens B, Goldschmeding R, van Geffen AJ, Nous FM, van Kuik J, van der Weide P, Klöpping C, Kirkels JH, Lahpor JR, Doevendans PA, de Jonge N, de Weger RA. Circulating growth differentiation factor-15 correlates with myocardial fibrosis in patients with non-ischaemic dilated cardiomyopathy and decreases rapidly after left ventricular assist device support. Eur J Heart Fail. 2012; 14:1249–56. 10.1093/eurjhf/hfs120 22843564

[127] Tromp J, Westenbrink BD, Ouwerkerk W, van Veldhuisen DJ, Samani NJ, Ponikowski P, Metra M, Anker SD, Cleland JG, Dickstein K, Filippatos G, van der Harst P, Lang CC, et al. Identifying Pathophysiological Mechanisms in Heart Failure With Reduced Versus Preserved Ejection Fraction. J Am Coll Cardiol. 2018; 72:1081–90. 10.1016/j.jacc.2018.06.050 30165978

[128] Chan MM, Santhanakrishnan R, Chong JP, Chen Z, Tai BC, Liew OW, Ng TP, Ling LH, Sim D, Leong KT, Yeo PS, Ong HY, Jaufeerally F, et al. Growth differentiation factor 15 in heart failure with preserved vs. reduced ejection fraction. Eur J Heart Fail. 2016; 18:81–88. 10.1002/ejhf.431 26497848

[129] Salminen A, Kaarniranta K, Kauppinen A. Regulation of longevity by FGF21: interaction between energy metabolism and stress responses. Ageing Res Rev. 2017; 37:79–93. 10.1016/j.arr.2017.05.004 28552719

[130] Kumar KG, Trevaskis JL, Lam DD, Sutton GM, Koza RA, Chouljenko VN, Kousoulas KG, Rogers PM, Kesterson RA, Thearle M, Ferrante AW Jr, Mynatt RL, Burris TP, et al. Identification of adropin as a secreted factor linking dietary macronutrient intake with energy homeostasis and lipid metabolism. Cell Metab. 2008; 8:468–81. 10.1016/j.cmet.2008.10.011 19041763 PMC2746325

[131] Kalkan AK, Cakmak HA, Erturk M, Kalkan KE, Uzun F, Tasbulak O, Diker VO, Aydin S, Celik A. Adropin and Irisin in Patients with Cardiac Cachexia. Arq Bras Cardiol. 2018; 111:39–47. 10.5935/abc.20180109 29972412 PMC6078358

[132] Thapa D, Xie B, Zhang M, Stoner MW, Manning JR, Huckestein BR, Edmunds LR, Mullett SJ, McTiernan CF, Wendell SG, Jurczak MJ, Scott I. Adropin treatment restores cardiac glucose oxidation in pre-diabetic obese mice. J Mol Cell Cardiol. 2019; 129:174–78. 10.1016/j.yjmcc.2019.02.012 30822408 PMC6486841

[133] Thapa D, Stoner MW, Zhang M, Xie B, Manning JR, Guimaraes D, Shiva S, Jurczak MJ, Scott I. Adropin regulates pyruvate dehydrogenase in cardiac cells via a novel GPCR-MAPK-PDK4 signaling pathway. Redox Biol. 2018; 18:25–32. 10.1016/j.redox.2018.06.003 29909017 PMC6008287

[134] Kwon OS, Andtbacka RH, Hyngstrom JR, Richardson RS. Vasodilatory function in human skeletal muscle feed arteries with advancing age: the role of adropin. J Physiol. 2019; 597:1791–804. 10.1113/JP277410 30690728 PMC6441888

[135] Lovren F, Pan Y, Quan A, Singh KK, Shukla PC, Gupta M, Al-Omran M, Teoh H, Verma S. Adropin is a novel regulator of endothelial function. Circulation. 2010 (11 Suppl); 122:S185–92. 10.1161/CIRCULATIONAHA.109.931782 20837912

[136] Yosaee S, Soltani S, Sekhavati E, Jazayeri S. Adropin- A Novel Biomarker of Heart Disease: A Systematic Review Article. Iran J Public Health. 2016; 45:1568–76. 28053922 PMC5207097

[137] Boström P, Wu J, Jedrychowski MP, Korde A, Ye L, Lo JC, Rasbach KA, Boström EA, Choi JH, Long JZ, Kajimura S, Zingaretti MC, Vind BF, et al. A PGC1-α-dependent myokine that drives brown-fat-like development of white fat and thermogenesis. Nature. 2012; 481:463–68. 10.1038/nature10777 22237023 PMC3522098

[138] Tsuchiya Y, Ando D, Goto K, Kiuchi M, Yamakita M, Koyama K. High-intensity exercise causes greater irisin response compared with low-intensity exercise under similar energy consumption. Tohoku J Exp Med. 2014; 233:135–40. 10.1620/tjem.233.135 24910199

[139] Aydin S, Kuloglu T, Aydin S, Eren MN, Celik A, Yilmaz M, Kalayci M, Sahin İ, Gungor O, Gurel A, Ogeturk M, Dabak O. Cardiac, skeletal muscle and serum irisin responses to with or without water exercise in young and old male rats: cardiac muscle produces more irisin than skeletal muscle. Peptides. 2014; 52:68–73. 10.1016/j.peptides.2013.11.024 24345335

[140] Hsieh IC, Ho MY, Wen MS, Chen CC, Hsieh MJ, Lin CP, Yeh JK, Tsai ML, Yang CH, Wu VC, Hung KC, Wang CC, Wang CY. Serum irisin levels are associated with adverse cardiovascular outcomes in patients with acute myocardial infarction. Int J Cardiol. 2018; 261:12–17. 10.1016/j.ijcard.2017.11.072 29657036

[141] Li R, Wang X, Wu S, Wu Y, Chen H, Xin J, Li H, Lan J, Xue K, Li X, Zhuo C, He J, Tang CS, Jiang W. Irisin ameliorates angiotensin II-induced cardiomyocyte apoptosis through autophagy. J Cell Physiol. 2019; 234:17578–88. 10.1002/jcp.28382 30793300

[142] Silvestrini A, Bruno C, Vergani E, Venuti A, Favuzzi AM, Guidi F, Nicolotti N, Meucci E, Mordente A, Mancini A. Circulating irisin levels in heart failure with preserved or reduced ejection fraction: A pilot study. PLoS One. 2019; 14:e0210320. 10.1371/journal.pone.0210320 30657767 PMC6338355

[143] Yoneda T, Benedetti C, Urano F, Clark SG, Harding HP, Ron D. Compartment-specific perturbation of protein handling activates genes encoding mitochondrial chaperones. J Cell Sci. 2004; 117:4055–66. 10.1242/jcs.01275 15280428

[144] Prokisch H, Scharfe C, Camp DG 2nd, Xiao W, David L, Andreoli C, Monroe ME, Moore RJ, Gritsenko MA, Kozany C, Hixson KK, Mottaz HM, Zischka H, et al. Integrative analysis of the mitochondrial proteome in yeast. PLoS Biol. 2004; 2:e160. 10.1371/journal.pbio.0020160 15208715 PMC423137

[145] Chacinska A, Koehler CM, Milenkovic D, Lithgow T, Pfanner N. Importing mitochondrial proteins: machineries and mechanisms. Cell. 2009; 138:628–44. 10.1016/j.cell.2009.08.005 19703392 PMC4099469

[146] Zhao Q, Wang J, Levichkin IV, Stasinopoulos S, Ryan MT, Hoogenraad NJ. A mitochondrial specific stress response in mammalian cells. EMBO J. 2002; 21:4411–19. 10.1093/emboj/cdf445 12198143 PMC126185

[147] Wu Z, Senchuk MM, Dues DJ, Johnson BK, Cooper JF, Lew L, Machiela E, Schaar CE, DeJonge H, Blackwell TK, Van Raamsdonk JM. Mitochondrial unfolded protein response transcription factor ATFS-1 promotes longevity in a long-lived mitochondrial mutant through activation of stress response pathways. BMC Biol. 2018; 16:147. 10.1186/s12915-018-0615-3 30563508 PMC6298126

[148] Shpilka T, Haynes CM. The mitochondrial UPR: mechanisms, physiological functions and implications in ageing. Nat Rev Mol Cell Biol. 2018; 19:109–20. 10.1038/nrm.2017.110 29165426

[149] Wrobel L, Topf U, Bragoszewski P, Wiese S, Sztolsztener ME, Oeljeklaus S, Varabyova A, Lirski M, Chroscicki P, Mroczek S, Januszewicz E, Dziembowski A, Koblowska M, et al. Mistargeted mitochondrial proteins activate a proteostatic response in the cytosol. Nature. 2015; 524:485–88. 10.1038/nature14951 26245374

[150] Nargund AM, Pellegrino MW, Fiorese CJ, Baker BM, Haynes CM. Mitochondrial import efficiency of ATFS-1 regulates mitochondrial UPR activation. Science. 2012; 337:587–90. 10.1126/science.1223560 22700657 PMC3518298

[151] Seli E, Wang T, Horvath TL. Mitochondrial unfolded protein response: a stress response with implications for fertility and reproductive aging. Fertil Steril. 2019; 111:197–204. 10.1016/j.fertnstert.2018.11.048 30691623

[152] Mohrin M, Shin J, Liu Y, Brown K, Luo H, Xi Y, Haynes CM, Chen D. Stem cell aging. A mitochondrial UPR-mediated metabolic checkpoint regulates hematopoietic stem cell aging. Science. 2015; 347:1374–77. 10.1126/science.aaa2361 25792330 PMC4447312

[153] Nargund AM, Fiorese CJ, Pellegrino MW, Deng P, Haynes CM. Mitochondrial and nuclear accumulation of the transcription factor ATFS-1 promotes OXPHOS recovery during the UPR(mt). Mol Cell. 2015; 58:123–33. 10.1016/j.molcel.2015.02.008 25773600 PMC4385436

[154] Lamech LT, Haynes CM. The unpredictability of prolonged activation of stress response pathways. J Cell Biol. 2015; 209:781–87. 10.1083/jcb.201503107 26101215 PMC4477854

[155] Nambi V, Liu X, Chambless LE, de Lemos JA, Virani SS, Agarwal S, Boerwinkle E, Hoogeveen RC, Aguilar D, Astor BC, Srinivas PR, Deswal A, Mosley TH, et al. Troponin T and N-terminal pro-B-type natriuretic peptide: a biomarker approach to predict heart failure risk—the atherosclerosis risk in communities study. Clin Chem. 2013; 59:1802–10. 10.1373/clinchem.2013.203638 24036936 PMC4208068

[156] Zhang H, Ryu D, Wu Y, Gariani K, Wang X, Luan P, D’Amico D, Ropelle ER, Lutolf MP, Aebersold R, Schoonjans K, Menzies KJ, Auwerx J. NAD⁺ repletion improves mitochondrial and stem cell function and enhances life span in mice. Science. 2016; 352:1436–43. 10.1126/science.aaf2693 27127236

[157] Xu M, Xue RQ, Lu Y, Yong SY, Wu Q, Cui YL, Zuo XT, Yu XJ, Zhao M, Zang WJ. Choline ameliorates cardiac hypertrophy by regulating metabolic remodelling and UPRmt through SIRT3-AMPK pathway. Cardiovasc Res. 2019; 115:530–45. 10.1093/cvr/cvy217 30165480

[158] Lin YF, Schulz AM, Pellegrino MW, Lu Y, Shaham S, Haynes CM. Maintenance and propagation of a deleterious mitochondrial genome by the mitochondrial unfolded protein response. Nature. 2016; 533:416–19. 10.1038/nature17989 27135930 PMC4873342

[159] Martinez BA, Petersen DA, Gaeta AL, Stanley SP, Caldwell GA, Caldwell KA. Dysregulation of the Mitochondrial Unfolded Protein Response Induces Non-Apoptotic Dopaminergic Neurodegeneration in *C. elegans* Models of Parkinson’s Disease. J Neurosci. 2017; 37:11085–100. 10.1523/JNEUROSCI.1294-17.2017 29030433 PMC5688521

[160] Steele HE, Horvath R, Lyon JJ, Chinnery PF. Monitoring clinical progression with mitochondrial disease biomarkers. Brain. 2017; 140:2530–40. 10.1093/brain/awx168 28969370 PMC5841218

[161] Robinson BH. Lactic acidemia and mitochondrial disease. Mol Genet Metab. 2006; 89:3–13. 10.1016/j.ymgme.2006.05.015 16854608

[162] Milne GL, Musiek ES, Morrow JD. F2-isoprostanes as markers of oxidative stress in vivo: an overview. Biomarkers. 2005 (Suppl 1); 10:S10–23. 10.1080/13547500500216546 16298907

[163] Davis RL, Liang C, Edema-Hildebrand F, Riley C, Needham M, Sue CM. Fibroblast growth factor 21 is a sensitive biomarker of mitochondrial disease. Neurology. 2013; 81:1819–26. 10.1212/01.wnl.0000436068.43384.ef 24142477

[164] Quirós PM, Mottis A, Auwerx J. Mitonuclear communication in homeostasis and stress. Nat Rev Mol Cell Biol. 2016; 17:213–26. 10.1038/nrm.2016.23 26956194

[165] Tezze C, Romanello V, Desbats MA, Fadini GP, Albiero M, Favaro G, Ciciliot S, Soriano ME, Morbidoni V, Cerqua C, Loefler S, Kern H, Franceschi C, et al. Age-Associated Loss of OPA1 in Muscle Impacts Muscle Mass, Metabolic Homeostasis, Systemic Inflammation, and Epithelial Senescence. Cell Metab. 2017; 25:1374–89.e6. 10.1016/j.cmet.2017.04.021 28552492 PMC5462533

[166] Ost M, Coleman V, Voigt A, van Schothorst EM, Keipert S, van der Stelt I, Ringel S, Graja A, Ambrosi T, Kipp AP, Jastroch M, Schulz TJ, Keijer J, Klaus S. Muscle mitochondrial stress adaptation operates independently of endogenous FGF21 action. Mol Metab. 2015; 5:79–90. 10.1016/j.molmet.2015.11.002 26909316 PMC4735627

[167] Adela R, Banerjee SK. GDF-15 as a Target and Biomarker for Diabetes and Cardiovascular Diseases: A Translational Prospective. J Diabetes Res. 2015; 2015:490842. 10.1155/2015/490842 26273671 PMC4530250

[168] Fisher FM, Chui PC, Antonellis PJ, Bina HA, Kharitonenkov A, Flier JS, Maratos-Flier E. Obesity is a fibroblast growth factor 21 (FGF21)-resistant state. Diabetes. 2010; 59:2781–89. 10.2337/db10-0193 20682689 PMC2963536

[169] Díaz-Delfín J, Hondares E, Iglesias R, Giralt M, Caelles C, Villarroya F. TNF-α represses β-Klotho expression and impairs FGF21 action in adipose cells: involvement of JNK1 in the FGF21 pathway. Endocrinology. 2012; 153:4238–45. 10.1210/en.2012-1193 22778214

[170] Tanajak P, Sa-Nguanmoo P, Wang X, Liang G, Li X, Jiang C, Chattipakorn SC, Chattipakorn N. Fibroblast growth factor 21 (FGF21) therapy attenuates left ventricular dysfunction and metabolic disturbance by improving FGF21 sensitivity, cardiac mitochondrial redox homoeostasis and structural changes in pre-diabetic rats. Acta Physiol (Oxf). 2016; 217:287–99. 10.1111/apha.12698 27119620

[171] Markan KR, Naber MC, Small SM, Peltekian L, Kessler RL, Potthoff MJ. FGF21 resistance is not mediated by downregulation of beta-klotho expression in white adipose tissue. Mol Metab. 2017; 6:602–10. 10.1016/j.molmet.2017.03.009 28580290 PMC5444074

[172] Cops J, Haesen S, De Moor B, Mullens W, Hansen D. Current animal models for the study of congestion in heart failure: an overview. Heart Fail Rev. 2019; 24:387–97. 10.1007/s10741-018-9762-4 30612214 PMC6476831

[173] Batlle M, Castillo N, Alcarraz A, Sarvari S, Sangüesa G, Cristóbal H, García de Frutos P, Sitges M, Mont L, Guasch E. Axl expression is increased in early stages of left ventricular remodeling in an animal model with pressure-overload. PLoS One. 2019; 14:e0217926. 10.1371/journal.pone.0217926 31181097 PMC6557565

[174] McGill MR, Sharpe MR, Williams CD, Taha M, Curry SC, Jaeschke H. The mechanism underlying acetaminophen-induced hepatotoxicity in humans and mice involves mitochondrial damage and nuclear DNA fragmentation. J Clin Invest. 2012; 122:1574–83. 10.1172/JCI59755 22378043 PMC3314460

[175] Chou RH, Huang PH, Hsu CY, Chang CC, Leu HB, Huang CC, Chen JW, Lin SJ. Circulating Fibroblast Growth Factor 21 is Associated with Diastolic Dysfunction in Heart Failure Patients with Preserved Ejection Fraction. Sci Rep. 2016; 6:33953. 10.1038/srep33953 27650781 PMC5030655

[176] Lymperopoulos A, Rengo G, Koch WJ. Adrenergic nervous system in heart failure: pathophysiology and therapy. Circ Res. 2013; 113:739–53. 10.1161/CIRCRESAHA.113.300308 23989716 PMC3843360

[177] Xu XY, Nie Y, Wang FF, Bai Y, Lv ZZ, Zhang YY, Li ZJ, Gao W. Growth differentiation factor (GDF)-15 blocks norepinephrine-induced myocardial hypertrophy via a novel pathway involving inhibition of epidermal growth factor receptor transactivation. J Biol Chem. 2014; 289:10084–94. 10.1074/jbc.M113.516278 24554716 PMC3974979

[178] Li S, Zhu Z, Xue M, Yi X, Liang J, Niu C, Chen G, Shen Y, Zhang H, Zheng J, Zhao C, Liang Y, Cong W, et al. Fibroblast growth factor 21 protects the heart from angiotensin II-induced cardiac hypertrophy and dysfunction via SIRT1. Biochim Biophys Acta Mol Basis Dis. 2019; 1865:1241–52. 10.1016/j.bbadis.2019.01.019 30677512

[179] Cuevas-Ramos D, Almeda-Valdés P, Meza-Arana CE, Brito-Córdova G, Gómez-Pérez FJ, Mehta R, Oseguera-Moguel J, Aguilar-Salinas CA. Exercise increases serum fibroblast growth factor 21 (FGF21) levels. PLoS One. 2012; 7:e38022. 10.1371/journal.pone.0038022 22701542 PMC3365112

[180] Chen B, Lu D, Fu Y, Zhang J, Huang X, Cao S, Xu D, Bin J, Kitakaze M, Huang Q, Liao Y. Olmesartan prevents cardiac rupture in mice with myocardial infarction by modulating growth differentiation factor 15 and p53. Br J Pharmacol. 2014; 171:3741–53. 10.1111/bph.12736 24749959 PMC4128070

[181] Li RL, Wu SS, Wu Y, Wang XX, Chen HY, Xin JJ, Li H, Lan J, Xue KY, Li X, Zhuo CL, Cai YY, He JH, et al. Irisin alleviates pressure overload-induced cardiac hypertrophy by inducing protective autophagy via mTOR-independent activation of the AMPK-ULK1 pathway. J Mol Cell Cardiol. 2018; 121:242–55. 10.1016/j.yjmcc.2018.07.250 30053525

[182] Kharitonenkov A, DiMarchi R. Fibroblast growth factor 21 night watch: advances and uncertainties in the field. J Intern Med. 2017; 281:233–46. 10.1111/joim.12580 27878865

[183] Kahn BB. Adipose Tissue, Inter-Organ Communication, and the Path to Type 2 Diabetes: The 2016 Banting Medal for Scientific Achievement Lecture. Diabetes. 2019; 68:3–14. 10.2337/dbi18-0035 30573674 PMC6302542

[184] Droujinine IA, Perrimon N. Interorgan Communication Pathways in Physiology: focus on Drosophila. Annu Rev Genet. 2016; 50:539–70. 10.1146/annurev-genet-121415-122024 27732790 PMC5506552

